# Nitrogen Addition Increases the Sensitivity of Photosynthesis to Drought and Re-watering Differentially in C_3_ Versus C_4_ Grass Species

**DOI:** 10.3389/fpls.2019.00815

**Published:** 2019-07-03

**Authors:** Shangzhi Zhong, Yueqiao Xu, Bo Meng, Michael E. Loik, Jian-Ying Ma, Wei Sun

**Affiliations:** ^1^Key Laboratory of Vegetation Ecology, Ministry of Education, Institute of Grassland Science, Northeast Normal University, Changchun, China; ^2^Department of Environmental Studies, University of California, Santa Cruz, Santa Cruz, CA, United States; ^3^Key Laboratory of Biogeography and Bioresources in Arid Land, Xinjiang Institute of Ecology and Geography, Chinese Academy of Sciences, Ürümqi, China

**Keywords:** extreme climate, N deposition, recovery extent and rate asymmetry, resistance, resilience, water pulse

## Abstract

Global change factors, such as variation in precipitation regimes and nitrogen (N) deposition, are likely to occur simultaneously and may have profound impacts on the relative abundance of grasses differing in functional traits, such as C_3_ and C_4_ species. We conducted an extreme drought and re-watering experiment to understand differences in the resistance and recovery abilities of C_3_ and C_4_ grasses under different N deposition scenarios. A C_3_ perennial grass (*Leymus chinensis*) and two C_4_ grasses (annual species *Chloris virgata* and perennial species *Hemarthria altissima*) that co-occur in Northeast China were selected as experimental plants. For both C_3_ and C_4_ grasses, N addition caused a strong increase in biomass and resulted in more severe drought stress, leading to a change in the dominant photosynthetic limitation during the drought periods. Although N addition increased antioxidant enzyme activities and protective solute concentrations, the carbon fixing capacity did not fully recover to pre-drought levels by the end of the re-watering period. N addition resulted in lower resilience under the drought conditions and lower resistance at the end of the re-watering. However, N addition led to faster recovery of photosynthesis, especially in the C_3_ grass, which indicate that the effect of N addition on photosynthesis during drought was asymmetric, especially in the plants with different photosynthetic nitrogen use efficiency (PNUE). These findings demonstrated that nitrogen deposition may significant alter the susceptibility of C_3_ and C_4_ grass species to drought stress and re-watering, highlighting the asymmetry between resistance and resilience and to improve our understanding about plant responses to climate change.

## Introduction

Globally, the intensification of the hydrologic cycle (Knapp et al., 2008; Smith, 2011; IPCC, 2013; Knapp et al., 2015) is expected to increase the frequency and magnitude of climate extremes, such as severe drought and intense rainfall (Karl et al., 1995; Hoerling and Kumar, 2003; Reichstein et al., 2013). These extreme climate events are likely to interact with chronic environmental changes, such as N deposition (Liu et al., 2011, 2013; Zhu et al., 2016), to alter key aspects of ecosystem carbon cycling, such as photosynthetic carbon assimilation (Xia and Wan, 2008; Xu et al., 2013; Wang et al., 2015; Shi et al., 2018). C_3_ and C_4_ plants may respond differently to altered precipitation and N deposition due to functional differences in anatomical structure, photosynthetic processes, responses to temperature, and water and N use efficiency (Long, 1999; Yuan et al., 2007; Niu et al., 2008a; Ripley et al., 2010; Taylor et al., 2014). Such differences could cause substantial changes in vegetation composition and ecosystem functions (Still et al., 2003).

Plants with the C_4_ enzyme pathway for concentrating CO_2_ can reduce stomatal aperture while fixing CO_2_ at rates equal to or greater than C_3_ species; thus, C_4_ species have higher water-use efficiency (WUE) than C_3_ species (Pearcy and Ehleringer, 1984; Taylor et al., 2014). Based on relative performance of C_3_ and C_4_ photosynthesis, C_4_ species are expected to outperform C_3_ species in warm habitats and conditions that promote stomatal closure (drought, warm temperatures, high salinity, and low humidity) (Taiz and Zeiger, 1991; Raven et al., 1992; Haxeltine and Prentice, 1996). Some previous studies have reported that C_4_ species can maintain high WUE under drought conditions and thus maintain their photosynthetic advantage over C_3_ species (Taylor et al., 2014). However, other studies found that leaf carbon assimilation in C_4_ species is sensitive to variation in soil water content, such that they can sometimes even lose their photosynthetic advantage (Ripley et al., 2007, 2010; Ibrahim et al., 2008; Taylor et al., 2011).

The inhibitory effects of drought on photosynthesis can be associated with low CO_2_ availability within the stroma of chloroplasts caused by diffusion limitations through the stomata and the mesophyll (Flexas et al., 2004), the alteration of enzymatic carbon assimilation (Lawlor, 2002; Lawlor and Cornic, 2002), and phloem transport limitations (Mencuccini and Hölttä, 2010). Stomatal closure is an early response to drought and an efficient way to reduce water loss when drought is not too severe; however, it limits CO_2_ diffusion into the leaves for photosynthesis (Cornic et al., 2000). Changes in mesophyll conductance (*g*_m_) may also play an important role in the drought-induced reduction of leaf carbon supply for assimilation (Grassi and Magnani, 2005). The sum of both stomatal and mesophyll limitations represents the diffusional limitations of the key photosynthetic substrate, CO_2_. Inhibition of metabolic activities occurs with prolonged periods of drought stress (Parry et al., 2002). Biochemical or metabolic (non-stomatal) limitations of photosynthesis under drought stress include reductions in carboxylation efficiency (CE), a decline in the maximum velocity for carboxylation of Rubisco (V_cmax_), the maximal phosphoenolpyruvate carboxylation rate (V_pmax_), ribulose-1,5-bisphosphate (RuBP) regeneration capacity mediated by maximum electron rate (J_max_), and decreases in activities of Photosystem II (PSII) and Rubisco (Giardi et al., 1996; Pelloux et al., 2001; Parry et al., 2002; Jia and Gray, 2004; Peña-Rojas et al., 2004; Flexas et al., 2006).

In addition to the rate and degree of photosynthetic decline during soil water depletion, the carbon balance of a plant exposed to water stress may also depend on the rate and degree of photosynthetic recovery after re-watering. Because of the greater magnitude of metabolic limitations in C_4_ species under severe drought, C_4_ species are typically more sensitive to drought than C_3_ species, and their recovery from drought is often slower (Ripley et al., 2010). Whereas many studies have addressed different aspects of photosynthetic limitations during drought, analyses of photosynthetic limitations during recovery from water stress are relatively few (Miyashita et al., 2005; Flexas et al., 2006).

Drought resistance and resilience in the context of leaf photosynthesis may be influenced by other factors, such as osmotic adjustment ability, antioxidant enzyme activity, other thermal dissipation processes for PSII (e.g., the xanthophyll cycle), and N availability. These are crucial processes in plant responses to drought (Morgan, 1984; Chaves et al., 2003) and important in the avoidance of oxidative stress (Smirnoff, 1998; Dat et al., 2000; Sharma et al., 2012). C_3_ and C_4_ plants may differ in the metabolism of activated oxygen species (Zhang and Kirkham, 1996) and osmotic adjustment ability, which will change the abilities of C_3_ and C_4_ species to acclimate to dramatic variation in water conditions.

Using a pot experiment, we subjected a controlled drought to one C_3_ (*Leymus chinensis*) and two C_4_ (*Chloris virgata* and *Hemarthria altissima*) grass species that co-occur widely in the meadow steppe of Northeast China, followed by a re-watering treatment under both fertilized and unfertilized conditions. To assess differences in drought resistance and resilience between the C_3_ and C_4_ grasses, and the effects of N supply on these traits, we measured soil moisture, leaf water potential, leaf-level photosynthetic gas exchange parameters, CO_2_ dose-response (*A*-*C*_i_) curves, chlorophyll fluorescence, leaf antioxidant enzyme activity, and protective solute concentrations during the drought and re-watering periods. We hypothesized that (1) the C_4_ grasses would lose their advantages in WUE, relative to the C_3_ grass, under drought conditions; (2) stomatal limitation plays a more important role for drought impacts on leaf carbon assimilation in C_3_ grass than in C_4_ grasses; therefore, the C_3_ grass would recover more quickly than the C_4_ grasses after rehydration; and (3) compared to the C_4_ grasses, the C_3_ grass would likely benefit, in terms of drought resistance and resilience, from increased N supply, due to the differences in photosynthetic nitrogen use efficiency (PNUE) between C_3_ and C_4_ species.

## Materials and Methods

### Study Site and Experimental Design

The experiment was conducted in an open greenhouse at the Grassland Ecological Research Station of the Northeast Normal University, China (44°32′N, 123°40′E, 138–167 masl). The research area has a semi-arid, continental climate with a mean annual temperature of 6.3°C (1950–2014) and annual precipitation ranges from 280 to 644 mm (1950–2014) with over 70% of the precipitation occurring from June to August (Zhong et al., 2017). Potential evapotranspiration is approximately three times that of the annual precipitation (Qu and Guo, 2003). Vegetation is dominated by *L. chinensis* (Poaceae) (Trin.) Tzvel. a C_3_ perennial rhizomatous grass; *Phragmites australis*, *C. virgata*, and *H. altissima* are also abundant (Wang et al., 2018). The soil is classified as chernozem, with soil organic carbon content of 2.0% and soil total nitrogen content of 0.15% (Wang et al., 2015).

One C_3_ perennial grass (*L. chinensis*) and two C_4_ grasses [*C. virgata* (Poaceae) Sw., an annual, and *H. altissima* (Poaceae) (Poir.) Stapf & C.E. Hubbard, a perennial] that co-occur in the Songnen meadow steppe were selected as experimental species. On 15th May, 2015, seedlings of *L. chinensis* and *H. altissima* were transplanted into plastic pots (23.5 cm in diameter and 20 cm in height) filled with chernozem soil (8 kg soil pot^–1^). For *C. virgata*, plants were germinated from seeds on 1st May and transplanted into the plastic pots on 15th May. In order to have enough leaves to do all the destructive sampling, all species were planted with five individuals per pot in monoculture. For soil nitrogen, there were unfertilized (N0) and fertilized (N10) treatments, applied in a completely randomized design. For the fertilized treatment, NH_4_NO_3_ and granular urea (inorganic nitrogen:organic nitrogen = 7:3) were added to each pot at a rate of 10 g N m^–2^ y^–1^. Other macro- and micro-nutrients (P, K, S, Zn, Cu, Mn, Mo, B, and Fe) were applied for all treatments to ensure that plant growth was not limited by nutrients other than N. Before the initiation of the drought treatment, all the transplanted plants were placed outside of the greenhouse and well-watered to ensure that plant growth was not limited by water. During the drought treatment (14th – 20th July), all the pots were placed under a transparent plastic shed to exclude natural precipitation. All the pots were watered to 70% of field capacity during the re-watering period (21st – 27th July). The measurements of leaf gas exchange and the collection of fresh leaf materials were conducted on 14th July (Day 1), 16th July (Day 3), 18th July (Day 5), 20th July (Day 7), and 27th July (Day 14). Timeline figure indicating the dates, age of plants and treatment is provided as Supplementary Information (Supplementary Figure 1). On the aforementioned sampling dates, mature leaves were sampled between 0900 and 1100 h for the determination of antioxidant enzyme activity, proline, soluble sugar, and starch content. The collected leaves were immediately flash frozen in liquid nitrogen and temporarily stored in a deep freezer (−80°C).

### Meteorological Data and Soil Water Content

Air temperature, photosynthetic photon flux density (PPFD), relative humidity, and vapor pressure deficit were obtained from an eddy flux tower approximately 2 km away from the experimental site. Volumetric soil water content (SWC-V) at 0–10 cm soil depth was measured using a time-domain reflectometry (TDR; TRIME-PICO32) probe (IMKO, Ettlingen, Germany) with single measurement mode and recorded by HD2 Hand-Measurement Device at three points in each pot.

### Leaf Gas Exchange

Leaf gas exchange parameters, including net CO_2_ assimilation rate (*A*), transpiration rate (*E*) and stomatal conductance to water vapor (*g*_s_), were measured on Day 1, 3, 5, and 7 during the drought period and Day 14 during the re-watering period, using an LI-6400 portable photosynthesis system (Li-Cor Biosciences, Lincoln, NE, United States). For each species, leaf photosynthesis measurements were conducted between 0900 and 1100 h on both unfertilized and fertilized plants (six replicates). In each pot, two of the upper-most fully expanded leaves (the 2nd or 3rd leaf from the top) were used for leaf gas exchange measurements. Inside of the leaf chamber, PPFD was set at 2000 μmol m^–2^ s^–1^, air temperature at 25°C and CO_2_ concentration at 400 ppm. As gas exchange rates change linearly along the length of the leaf, measuring at the center of the leaf provides an estimate of the integrated whole-leaf gas exchange rate. Leaf level intrinsic WUE (*A*/*g*_s_) and instantaneous WUE (*A*/*E*) were calculated as the ratio of net CO_2_ assimilation rate to stomatal conductance and transpiration rate, respectively.

The resistance and resilience of photosynthetic capacity were calculated as the percent loss of *A* (*PLA*) and the percent recovery of *A* (*PRA*), respectively:

$$PLA\left(\%\right)=\left(\frac{A_{i}-A_{d}}{A_{i}}\right)\times 100\%\text{ and }PRA\left(\%\right)=\left(\frac{A_{r}}{A_{i}}\right)\times 100\%$$

where *A*_i_, *A*_d_, and *A*_r_ represent *A* at the initial period of the drought treatment, the end of the drought treatment, and after the re-watering treatment, respectively.

The capacity for photosynthetic recovery (recovery rate of *A*) was calculated as:

$$\mathrm{Recovery}\,\mathrm{rate}\,\mathrm{of}\,A=\frac{A_{r}-A_{d}}{D_{r}}$$

where *A*_r_ and *A*_d_ represent *A* at the end of the re-watering treatment and at the end of the drought treatment, respectively, and *D*_r_ represents the length (days) of the re-watering treatment.

A leaf chamber fluorometer (Model Li-6400-40) was used to determine the chlorophyll fluorescence parameter *F*_V_/*F*_M_. Leaves comparable to those used for the gas exchange measurements were used for the chlorophyll fluorescence measurements and the measurements were performed before dawn to ensure full reduction of Photosystem II. A measuring light of about 0.5 μmol photon m^–2^ s^–1^ was set at a frequency of 600 Hz to determine the background fluorescence signal (*F*_o_). Then, a saturating flash of about 10000 μmol photon m^–2^ s^–1^ and duration of 0.8 s was applied for the estimation of the maximum fluorescence (*F*_m_). Leaf photochemical efficiency (maximum quantum efficiency of Photosystem II) was calculated as: *F*_V_/*F*_M_ = (*F*_m_–*F*_o_)/*F*_m_ (Galmés et al., 2007a).

### *A*/*C*_i_ Curves

The relationship between *A* and intercellular CO_2_ concentration (*C*_i_) was measured using the LI-6400 portable photosynthesis system over a range of external CO_2_ concentrations (*C*_a_) from 50 to 2000 μmol mol^–1^ (in the order 400, 300, 200, 150, 100, 80, 60, 400, 400, 600, 800, 1000, 1500, and 2000 μmol mol^–1^) on days 1, 3, 5, 7, and 14 of the drought/re-watering period. For each species, four pots were used for *A*/*C*_i_ curve measurements. For each pot, first non-apical and fully expanded leaves were measured for the carbon fixation capacity measurements. Leaf chamber environmental conditions included PPFD = 2000 μmol m^–2^ s^–1^, leaf temperature = 25°C, and vapor pressure deficit = approx. 1.3 kPa. For the C_3_ grass, CO_2_ response curves were analyzed using the models of Von Caemmerer (2000) and temperature corrections were performed using the equations from Bernacchi et al. (2001) and Bernacchi et al. (2003). For the C_4_ grasses, *A*/*C*_i_ curves were modeled according to Collatz et al. (1992). Custom made macros were built in Microsoft Office Excel and used to estimate parameters from the *A*/*C*_i_ curves.

### Photosynthetic Limitation

The drought-induced reduction in *A* (relative to the average value for well-watered plants) was attributed to relative stomatal limitation (R_SL_) and relative metabolic limitation (R_ML_), which were modified from the models proposed by Ripley et al. (2010):

$$R_{\mathrm{SL}}=\left(\frac{A_{\mathrm{Ci},x}-A_{\mathrm{Ca},x}}{A_{\mathrm{Ca},\mathrm{day1}}}\right)\times 100\mathrm{and}$$

$$R_{\mathrm{ML}}=\left(\frac{A_{\mathrm{Ca},\mathrm{day1}}-A_{\mathrm{Ci},x}}{A_{\mathrm{Ca},\mathrm{day1}}}\right)\times 100$$

where *A*_C__i__, x_ are the net carbon assimilation rates at an intercellular CO_2_ concentration of 400 ppm (assuming no stomatal limitation) and *A*_C__a__, x_ are the net carbon assimilation rates at an atmospheric CO_2_ concentration of 400 ppm on days *x* = 1, 5, and 7 of drought/re-watering period, respectively; *A*_C__a__,__day1_ is the net carbon assimilation rates at an atmospheric CO_2_ concentration of 400 ppm on day 1 of the drought/re-watering period. For all studies, the values of R_ML_ on day 1 cannot be calculated.

### Mid-Day Water Potential

On the days of gas exchange measurements, mid-day leaf water potential (Ψ_m__d_) was measured between 1100 and 1300 h using a pressure chamber (PMS Instruments, Corvallis, OR, United States). For each species, Ψ_md_ measurements were repeated six times for each treatment. In each pot, two of the upper-most fully expanded leaves (the 2nd or 3rd leaf from the top) were used for Ψ_md_ measurements.

### Determination of Solute Concentrations and Water Relations Parameters

Proline was quantified by the acid-ninhydrin procedure (Bates et al., 1973). Acid-ninhydrin was prepared by warming 1.25 g ninhydrin in 30 ml of glacial acetic acid and 20 ml of 6 M phosphoric acid, with agitation, until dissolved. Leaf samples (approximately 0.5 g) were homogenized with 3% sulphosalicylic acid (10 ml) and clarified by centrifugation (3500 × *g* for 10 min). The supernatant (2 ml) was mixed with 2 ml of acid-ninhydrin and glacial acetic acid, the mixture was oven incubated at 100°C for 1 h, and the reaction was finished in an ice bath. The reaction mixture was extracted with toluene (4 ml) and absorbance was read at 520 nm, using toluene for a blank. The proline concentration was determined from a standard curve and calculated on a fresh weight basis. Two replicates were measured for each sample, and their mean values were used for further analysis. Leaf soluble carbohydrates and starch concentration were measured according to the microplate enzymatic method (Zhao et al., 2010).

Leaf osmotic pressure (π) was calculated based on the van’t Hoff Relation at 25°C, which was performed using the equations from Loik and Nobel (1991) and Nobel (2001):

$$\pi =R\,T\,\Sigma_{C_{I}}$$

where *R* is the gas constant (8.314 J mol^–1^ K^–1^), *T* is temperature on the absolute scale and C_I_ is concentration of all osmotically active solotes (leaf proline and soluble carbohydrates).

### Antioxidant Enzymes

To assay the activities of catalase (CAT, EC 1.11.1.6), peroxidase (POD, EC 1.11.1.7), and superoxide dismutase (SOD, EC 1.15.1.1), leaf samples (approximately 0.5 g) were homogenized in an ice-cold mortar with 6 ml of ice-cold 50 mM sodium phosphate buffer (pH 7.0) containing 0.2 mM EDTA and 1% (w/v) polyvinylpyrrolidone (PVP). The homogenates were filtered and centrifuged at 4°C for 20 min at 15000 × *g*. The supernatant was collected and used for the assays of enzymatic activities (Zhang and Kirkham, 1996). Total SOD activity was determined by measuring its ability to inhibit the photochemical reduction of nitroblue tetrazolium (NBT), according to the method of Giannopolitis and Ries (1977) with some modifications (Chowdhury and Choudhuri, 1985; Zhang and Kirkham, 1996). Activities of CAT and POD were measured by the method of Chance and Maehly (1955). For CAT, the decomposition of H_2_O_2_ was monitored by a decline in absorbance at 240 nm [ε = 39.4 M^–1^ cm^–1^ Nelson and Kiesow (1972)] for 1 min. The reaction was initiated with the addition of enzyme extract (0.1 ml) to a 3 ml reaction mixture containing 50 mM phosphate buffer (pH 7.0) and 15 mM H_2_O_2_. For POD, the oxidation of guaiacol was measured by the increase in absorbance at 470 nm [ε = 26.6 m M^–1^ cm^–1^, Chance and Maehly (1955)] for 1 min. The assay contained 50 μl of 20 mM guaiacol, 2.83 ml of 10 mM phosphate buffer (pH 7.0), and 0.1 ml of enzyme extract. The reaction was started with the addition of 20 μl of 40 mM H_2_O_2_.

The protein contents of crude enzyme extracts were determined according to Bradford (1976) using bovine serum albumin (BSA) as a standard.

### Drought Induced Changes

Drought induced changes in X (e.g., soil water content, leaf mid-day water potential, and leaf osmotic pressure) was calculated as follows:

$$\text{Change in X during drought}=X_{\mathrm{day7}}-X_{\mathrm{day1}}$$

### Statistical Analysis

One-way analysis of variance (ANOVA) was used to assess drought and re-watering effects on Ψ_md_, volumetric soil water contents, *A*, *g*_s_, *E*, *A*/*g*_s_, *A*/*E*, *PLA*, *PRA*, recovery rate of *A*, relative stomatal and metabolic limitations of photosynthesis, SOD activity, CAT activity, and POD activity on both nitrogen treatments. *T*-tests were used to detect nitrogen treatment differences for the aforementioned indices. Two-way ANOVA was used to assess the drought/re-watering treatment (D and R, respectively) and nitrogen treatment (N0 vs. N10) effects on the maximum velocity for carboxylation of Rubisco (V_cmax_) and the maximal phosphoenolpyruvate carboxylation rate (V_pmax_) in *C. virgata* and *H. altissima*, V_cmax_, and the maximum electron rate (J_max_) in *L. chinensis*, and on proline, soluble sugar and starch content in all grasses. All analyses were carried out using SPSS software version 22 (SPSS Inc., IL, United States). Average values are reported as the arithmetic mean ± 1 SE.

## Results

### Meteorological Parameters

For the five measuring dates, there were minimal day-to-day differences in meteorological parameters, including diurnal mean air temperature, diurnal mean PPFD, diurnal mean relative humidity, and diurnal mean vapor pressure deficit (Supplementary Figure 2).

### Soil Water Content and Leaf Water Potential

**FIGURE 1:**
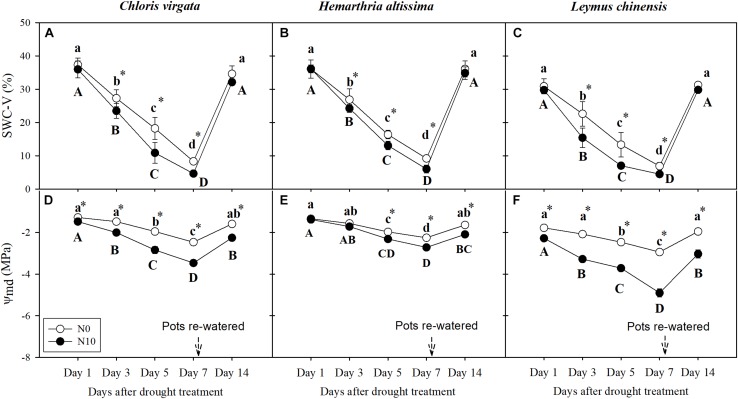
**(A–C)** Volumetric soil water content (SWC-V, %) and **(D–F)** leaf mid-day water potential (Ψ_md_; MPa) on days 1, 3, 5, 7, and 14 of the drought/ re-watering treatment in *Chloris virgata* (annual C_4_), *Hemarthria altissima* (perennial C_4_), and *Leymus chinensis* (perennial C_3_). Different lower-case letters and capital letters indicate significant differences between the measuring dates under the unfertilized (N0) treatment and the fertilized (N10) treatment, respectively. “^*^” represents significant differences between the N treatments (*P* < 0.05). Data are reported as the arithmetic mean ± 1 standard error (*n* = 6).

Soil water content (SWC) in both N treatments (N0 and N10) decreased during the drought period (Figures 1A–C). Notably, SWC in the fertilized pots was lower than in the unfertilized pots. Similar patterns were detected for leaf water potential (Ψ_md_) during the drought period, with Ψ_md_ in the fertilized plants significantly lower than in the unfertilized plants (Figures 1D–F). At the end of the re-watering period, Ψ_md_ in the unfertilized plants generally recovered to the pre-drought treatment level for all species. However, Ψ_md_ values in the fertilized plants at the end of the re-watering period were significantly lower than the pre-drought level for all species (Figures 1D–F).

### Leaf Gas Exchange During Drought and Re-watering

**FIGURE 2:**
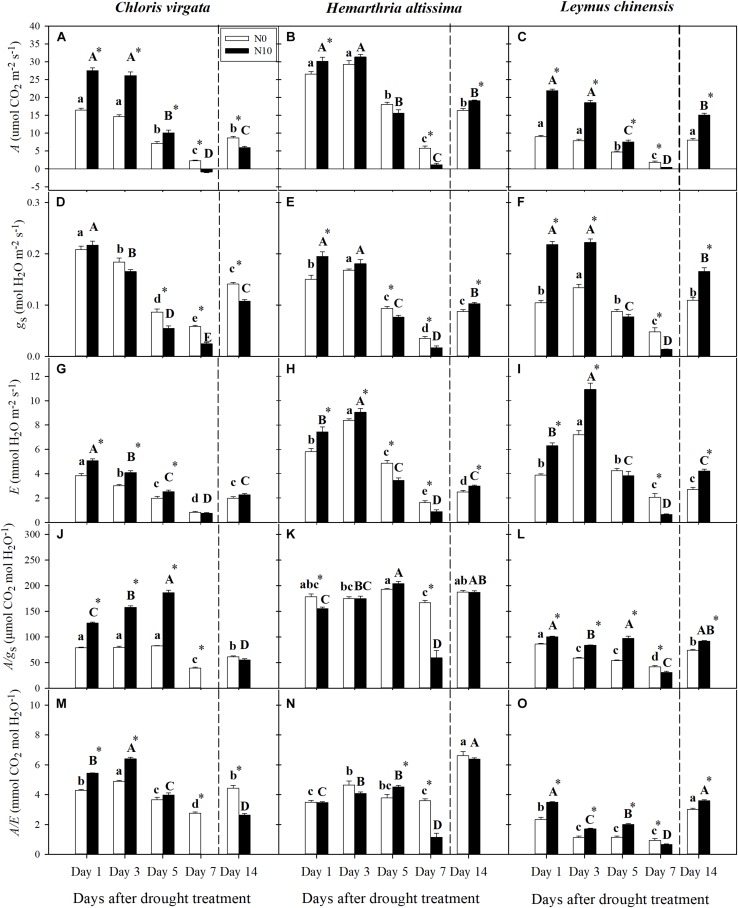
**(A–C)** Leaf net CO_2_ assimilation rate (*A*; μmol CO_2_ m^–2^ s^–1^), **(D–F)** stomatal conductance (*g*_s_; mol H_2_O m^–2^ s^–1^), **(G–I)** transpiration rate (E; mmol H_2_O m^–2^ s^–1^), **(J–L)** intrinsic water-use efficiency (WUE) (*A/g*_s_; μmol CO_2_ mol H_2_O^–1^), and **(M–O)** instantaneous WUE (*A/E*; mmol CO_2_ mol H_2_O^–1^) on days 1, 3, 5, 7, and 14 of the drought/re-watering treatment in *C. virgata* (annual C_4_), *H. altissima* (perennial C_4_), and *L. chinensis* (perennial C_3_). Different lower-case letters and capital letters indicate significant differences among the measuring dates under the unfertilized (N0) treatment and the fertilized (N10) treatment, respectively. “^*^” represents significant differences between the N treatments (*P* < 0.05). Data are reported as the arithmetic mean ± 1 standard error (*n* = 6). Vertical dashed lines denote the separation of drought and re-watering period.

For the three grass species, downregulation of net CO_2_ assimilation (*A*) occurred during the drought; however, these reductions were mainly apparent on day 5 and day 7, but not on day 3 (Figures 2A–C). Similar patterns were observed for stomatal conductance (*g*_s_) and transpiration rate *(E)* in *C. virgara*, as well as for *E* in the fertilized *H. altissima* and *L. chinensis* plants (Figures 2D–G). For *g*_s_ in the unfertilized *H. altissima* and *L. chinensis* plants and *E* in both fertilized and unfertilized *H. altissima* and *L. chinensis* plants, the values increased from day 1 to day 3 and decreased on day 5 and day 7 (Figures 2E,F,H,I). At the initial stage of the drought period (Day 1), there was significant upregulation of *A*, *g*_s_, and *E* in the fertilized grasses (compared to the unfertilized grasses); whereas the gas exchange parameters in the fertilized grasses were significantly downregulated compared to those of the unfertilized grasses at the end of drought period (Day 7) (Figures 2A–I). For *C. virgata* and *H. altissima*, there was not much variation in *A*/*g*_s_ and *A*/*E* detected during the drought period except on day 7 (Figures 2J,K,M,N). The WUE of *L. chinensis* gradually decreased during the drought period (From day 1 to day 7) (Figures 2L,O). For *C. virgata* and *L. chinensis*, there were significant decreases of *A*/*g*_s_ and *A*/*E* in the fertilized plants by comparison to those in the unfertilized plants (Figures 2J,L,M,O); whereas no N addition effects on *A*/*g*_s_ and *A*/*E* were detected for *H. altissima* (Figures 2K,N). At the end of the drought period (Day 7), *A*/*g*_s_ and *A*/*E* were lower in the fertilized plants by comparison to the unfertilized plants (Figures 2J–O).

At the end of the re-watering period (Day 14), *A*, *g*_s_, and *E* recovered to some extent for the three grass species for both the fertilized and unfertilized treatments, but the degree of recovery varied (Figures 2A–I). The values of *A* and *g*_s_ in the unfertilized *L. chinensis* plants recovered to their pre-drought treatment levels (Figures 2C,F). At the end of the re-watering period (Day 14), the values of leaf gas exchange parameters were significantly lower for unfertilized compared to the fertilized *H. altissima* and *L. chinensis* plants (Figures 2B,C,E,F,H,I). On the other hand, *A* and *g*_s_ were significantly higher for the unfertilized vs. fertilized *C. virgata* plants (Figures 2A,D), yet *E* did not differ between the unfertilized and fertilized plants (Figure 2G). The values of *A/g*_s_ and *A/E* under both the fertilized and unfertilized treatments were mostly restored in *H. altissima* and *L. chinensis* plants, but not in *C. virgata* plants (Figures 2J–O). Only *A*/*g*_s_ and *A*/*E* were significantly lower for the unfertilized compared to the fertilized *L. chinensis* plants (Figures 2L,O).

### Resistance and Resilience of Photosynthesis

**FIGURE 3:**
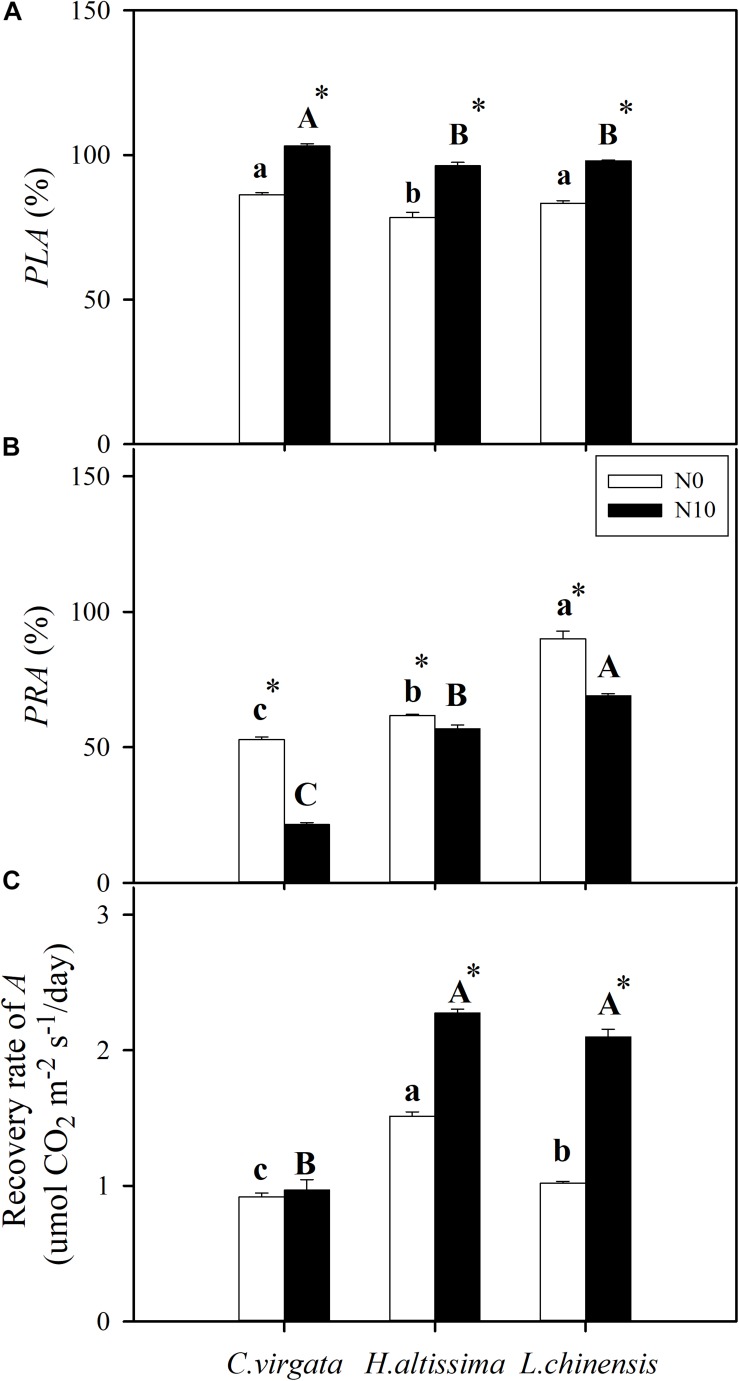
**(A)** Percentage loss of net CO_2_ assimilation rate (*PLA*), **(B)** percentage recovery of net CO_2_ assimilation rate (*PRA*), and **(C)** the recovery capability (recovery rate of *A*) in *C. virgata* (annual C_4_), *H. altissima* (perennial C_4_), and *L. chinensis* (perennial C_3_). Different lower-case letters and capital letters indicate significant differences among the studied grasses under the unfertilized (N0) treatment and the fertilized (N10) treatment, respectively. “^*^” represents significant differences between the N treatments (*P* < 0.05). Data are reported as the arithmetic mean ± 1 standard error (*n* = 6).

The percentage loss (*PLA*) and recovery (*PRA*) of net leaf-level CO_2_ assimilation (*A*) were used to assess drought resistance and resilience of photosynthetic capacity, respectively. For all grass species, *PLA* in the fertilized plants was significantly greater than in the unfertilized plants (Figure 3A). Without fertilization, the *PLA* of *H. altissima* was significantly lower than in the other two grasses. For fertilized plants, the *PLA* of *C. virgata* was significantly higher than the other two species (Figure 3A). During the re-watering period, *L. chinensis* had the highest *PRA* values; whereas *C. virgata* had the lowest *PRA* values for both the fertilized and unfertilized treatments (Figure 3B). Without fertilization, *H. altissima* had the highest recovery rate of *A*, followed by *L. chinensis* and *C. virgata* plants. For fertilized plants, the recovery rates of *A* in *H. altissima* and *L. chinensis* plants were greater than in *C. virgata* plants. The N addition treatment significantly increased the recovery rate of *A* in *H. altissima* and *L. chinensis*, but not in *C. virgata* plants (Figure 3C).

### Maximum Efficiency of Photosystem II

**FIGURE 4:**
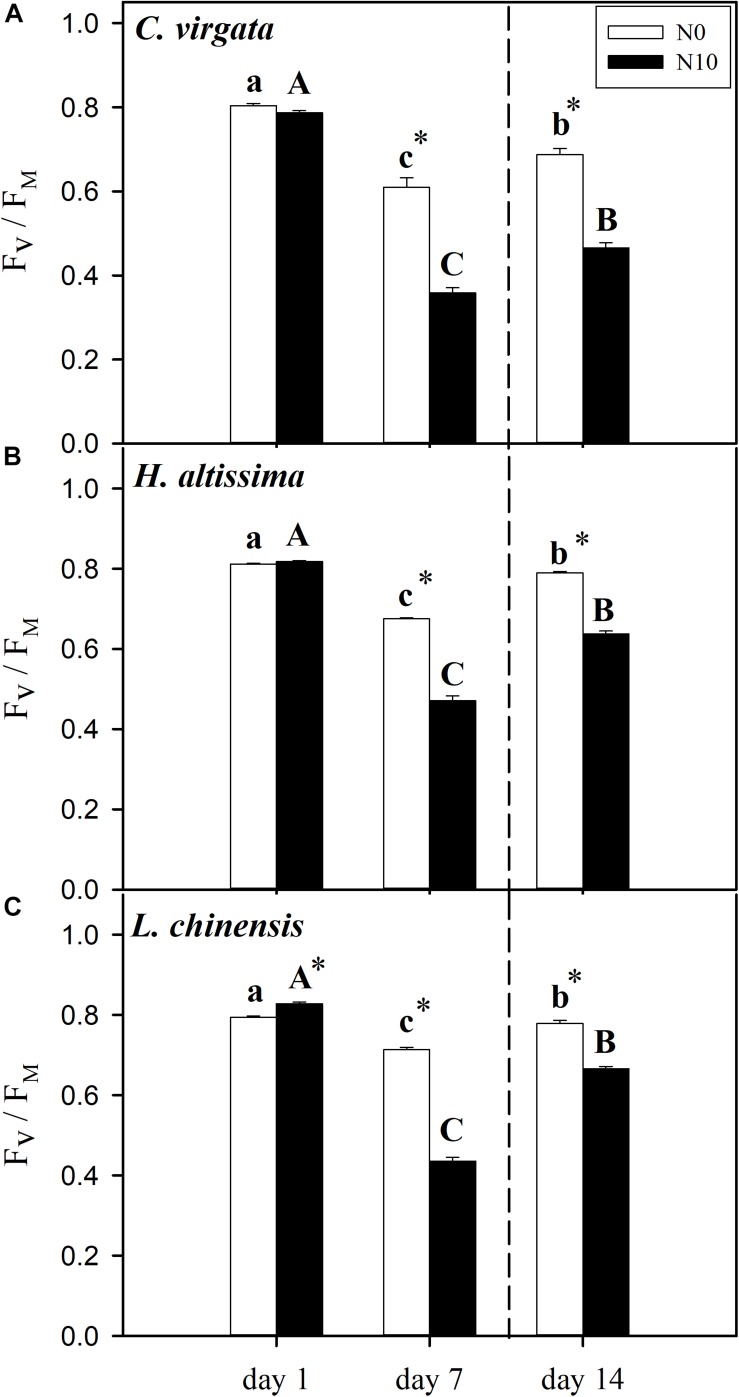
Leaf photochemical efficiency (maximum quantum efficiency of Photosystem II, *F*_V_/*F*_M_) in **(A)** *C. virgata* (annual C_4_), **(B)** *H. altissima* (perennial C_4_), and **(C)** *L. chinensis* (perennial C_3_) on days 1, 7, and 14 of the drought/re-watering treatment. Different lower-case letters and capital letters indicate significant differences among the measuring dates under the unfertilized (N0) treatment and the fertilized (N10) treatment, respectively. “^*^” represents significant differences between the N treatments (*P* < 0.05). Data are reported as the arithmetic mean ± 1 standard error (*n* = 6). Vertical dashed lines denote the separation of drought and re-watering period.

The values of leaf photochemical efficiency (*F*_V_/*F*_M_) on day 7 were lower than on day 1 for all grass species for both the fertilized and unfertilized treatments. Without fertilization, *F*_V_/*F*_M_ decreased from 0.80 ± 0.005, 0.81 ± 0.002, and 0.79 ± 0.003 on day 1 to 0.61 ± 0.021, 0.68 ± 0.002, and 0.71 ± 0.005 on day 7 for *C. virgata*, *H. altissima*, and *L. chinensis*, respectively. Moreover, significant downregulation of *F*_V_/*F*_M_ in the fertilized grasses was observed by comparison to those in the unfertilized grasses, from 0.79 ± 0.005, 0.82 ± 0.002, and 0.83 ± 0.003 on day 1 to 0.36 ± 0.012, 0.47 ± 0.011, and 0.44 ± 0.009 on day 7 for *C. virgata*, *H. altissima*, and *L. chinensis*, respectively (Figures 4A–C). At the end of the re-watering period, the *F*_V_/*F*_M_ values for both the C_3_ and C_4_ grasses were significantly lower than their pre-drought levels. For each grass species, considerable upregulation of *F*_V_/*F*_M_ in the unfertilized grasses was observed by comparison to those in the fertilized grasses. Comparing unfertilized to fertilized grasses, the *F*_V_/*F*_M_ values were 0.69 ± 0.014 versus 0.47 ± 0.011, 0.79 ± 0.003 versus 0.64 ± 0.006, and 0.78 ± 0.007 versus 0.67 ± 0.005 on day 14 for *C. virgata*, *H. altissima*, and *L. chinensis*, respectively (Figures 4A–C).

### Stomatal and Metabolic Limitation of Photosynthesis

**FIGURE 5:**
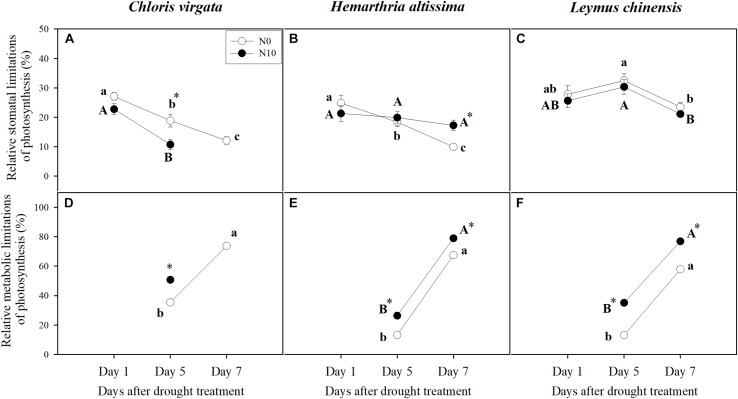
Relative stomatal and metabolic limitations of photosynthesis for **(A,D)** *C. virgata* (annual C_4_), **(B,E)** *H. altissima* (perennial C_4_), and **(C,F)** *L. chinensis* (perennial C_3_) on days 1, 5, and 7 of the drought treatment. Different lower-case letters and capital letters indicate significant differences among the measuring dates under the unfertilized (N0) treatment and the fertilized (N10) treatment, respectively. “^*^” represents significant differences between the different N addition treatments (*P* < 0.05). Data are reported as the arithmetic mean ± 1 standard error (*n* = 4).

Changes in *A* as a function of C_i_ were used to determine the relative stomatal limitation (R_SL_) and relative metabolic limitation (R_ML_) of photosynthesis during the drought treatment (Figure 5). Compared to the values on day 1, R_SL_ in *C. virgata* plants (fertilized and unfertilized) and *H. altissima* plants (unfertilized) decreased with time of drought (Figures 5A,B). However, no drought stress-induced variation in R_SL_ was detected for *L. chinensis* plants and fertilized *H. altissima* plants (Figures 5B,C). With the intensification of drought stress, R_ML_ increased for all grass species for both the fertilized and unfertilized treatments (Figures 5D–F). Meanwhile, R_ML_ in the fertilized grasses were significantly higher than in the unfertilized grasses (Figures 5D–F). R_SL_ and R_ML_ could not be calculated for the fertilized *C. virgata* plants on day 7 of the drought treatment due to negative values of *A* (Figures 5A,D).

The estimated V_cmax_, V_pmax_, and J_max_ values were significantly lower at the end of the drought treatment than their pre-drought values (Table 1). We were unable to estimate V_cmax_ and V_pmax_ for the fertilized *C. virgata* plants on day 7 because the extreme drought stress made it impossible to obtain complete *A*/*C*_i_ curves. During the drought period, significant N addition effects on V_cmax_, V_pmax_, and J_max_ were detected in *C. virgata* and *L. chinensis*, but not for *H. altissima*. For all grass species, V_cmax_, V_pmax_, and J_max_ in the fertilized grasses were significantly higher at the initial stage of drought (except *H. altissima*) and were significantly lower in the unfertilized grasses at the end of the drought treatment. At the end of the re-watering period, V_cmax_, V_pmax_, and J_max_ were significantly lower than prior to the onset of the drought treatment, with the exception of the unfertilized *L. chinensis* plants. The parameters were significantly influenced by N addition in *C. virgata* and *L. chinensis*, but not in *H. altissima*. The values of V_c__max_ and J_max_ in the fertilized *L. chinensis* plants were significantly higher than in the unfertilized *L. chinensis* plants; however, V_cmax_ and V_pmax_ in *C. virgata* plants under N addition were lower than in the absence of N addition (Table 1).

**TABLE 1:** Effects of the drought/re-watering treatment on maximum Rubisco capacity (V_c__max_), maximum PEP carboxylation rate (V_pmax_), and maximum rate of photosynthetic electron transport (J_max_) in *Chloris virgata* (annual C_4_), *Hemarthria altissima* (perennial C_4_), and *Leymus chinensis* (perennial C_3_) under both unfertilized (N0) and fertilized (N10) conditions.

**Species**			**Drought**			**Re-watering**	**Significance (D)**			**Significance (R)**		
			**Day 1**	**Day 5**	**Day 7**	**Day 14**	**D**	**N**	**D × N**	**R**	**N**	**R × N**
*Chloris virgata*	V_cmax_ (μmol m^–2^ s^–1^)	N0	23.41 ± 0.60	21.10 ± 2.06	7.06 ± 1.17	16.79 ± 1.52	^∗∗∗^	^∗∗∗^	^∗∗∗^	^∗∗∗^	^∗∗∗^	^∗∗∗^
		N10	51.30 ± 2.33	16.76 ± 1.80	−	10.82 ± 1.18						
	V_pmax_ (μmol m^–2^ s^–1^)	N0	24.65 ± 2.01	10.86 ± 0.26	8.28 ± 0.19	20.63 ± 0.93	^∗∗∗^	^∗∗∗^	^*^	^∗∗∗^	^∗∗∗^	^∗∗∗^
		N10	28.64 ± 0.15	17.95 ± 0.32	−	10.79 ± 1.44						
*Hemarthria altissima*	V_cmax_ (μmol m^–2^ s^–1^)	N0	44.51 ± 0.91	32.91 ± 2.67	23.77 ± 2.40	33.43 ± 0.98	^∗∗∗^	ns	^∗∗^	^∗∗∗^	ns	ns
		N10	47.01 ± 2.85	32.41 ± 1.73	14.18 ± 0.94	30.75 ± 2.39						
	V_pmax_ (μmol m^–2^ s^–1^)	N0	38.99 ± 3.00	28.49 ± 2.38	17.20 ± 2.53	19.60 ± 2.98	^∗∗∗^	ns	^∗∗∗^	^∗∗∗^	^∗∗∗^	ns
		N10	45.71 ± 2.58	37.82 ± 2.01	3.97 ± 1.07	33.44 ± 0.85						
*Leymus chinensis*	V_cmax_ (μmol m^–2^ s^–1^)	N0	63.88 ± 6.53	55.88 ± 8.29	21.65 ± 3.43	66.62 ± 6.30	^∗∗∗^	^∗∗∗^	^∗∗∗^	^∗∗∗^	^∗∗∗^	^∗∗∗^
		N10	158.47 ± 4.91	64.31 ± 6.91	11.03 ± 1.25	110.76 ± 7.39						
	J_max_ (μmol m^–2^ s^–1^)	N0	147.37 ± 6.25	105.94 ± 4.41	35.33 ± 0.30	137.52 ± 10.86	^∗∗∗^	^∗∗∗^	^∗∗∗^	^*^	^∗∗∗^	ns
		N10	220.40 ± 12.48	96.62 ± 5.50	17.47 ± 1.30	188.67 ± 15.42						

Results of two-way analysis of variance on the effects of drought (D), N addition (N), and their interactions (D **×** N), as well as re-watering (R), N, and their interactions (R **×** N) on V_*cmax*_, V_*pmax*_, and J_*max*_ are provided. Levels of significance are indicated as: ns – not significant, ^*^P < 0.05, ^∗∗^*P* < 0.01, and ^∗∗∗^*P* < 0.001. Data are reported as the arithmetic mean ± 1 standard error (*n* = 4).

### Solute Concentrations and Water Relations Parameters

Leaf proline content increased during drought for all species. The N addition strongly upregulated proline content (Table 2). Leaf soluble sugar content increased in the C_4_ grasses with the intensification of drought stress, whereas it decreased in the C_3_ grass. Leaf starch content gradually decreased from day 1 to day 7 (Table 2). Except for leaf starch content in *L. chinensis*, significant N addition effects were detected for soluble sugar and starch in all species. At the end of the re-watering period, leaf proline content remained at higher levels than the pre-drought values (Table 2). Compared to the values on day 1, significant differences were observed in leaf soluble sugar content and leaf starch content in *C. virgata* and *L. chinensis* plants, but not in *H. altissima* plants (Table 2).

**TABLE 2:** Effects of the drought/re-watering treatment on leaf proline content (μmol g^–1^), soluble sugar (mg g^–1^), and starch content (mg g^–1^) in *C. virgata* (annual C_4_), *H. altissima* (perennial C_4_), and *L. chinensis* (perennial C_3_).

**Species**			**Drought**				**Re-watering**	**Significance (D)**			**Significance (R)**		
			**Day 1**	**Day 3**	**Day 5**	**Day 7**	**Day 14**	**D**	**N**	**D × N**	**R**	**N**	**R × N**
*Chloris virgata*	Proline	N0	0.01 ± 0.01	0.11 ± 0.01	0.16 ± 0.01	0.32 ± 0.01	0.20 ± 0.01	^∗∗∗^	^∗∗∗^	^∗∗∗^	^∗∗∗^	^∗∗∗^	^∗∗∗^
	(μmol g^–1^)	N10	0.16 ± 0.01	0.24 ± 0.03	1.63 ± 0.14	10.52 ± 0.80	3.45 ± 0.78						
	Soluble sugar	N0	11.17 ± 0.65	15.22 ± 0.36	18.90 ± 0.50	23.11 ± 0.63	13.73 ± 0.61	^∗∗∗^	^∗∗∗^	^∗∗∗^	^∗∗^	^∗∗∗^	ns
	(mg g^–1^)	N10	14.03 ± 0.38	24.33 ± 0.88	29.34 ± 0.37	28.18 ± 0.82	16.78 ± 0.71						
	Starch	N0	41.06 ± 2.82	19.04 ± 1.15	10.36 ± 0.77	10.51 ± 0.27	27.94 ± 1.09	^∗∗∗^	^∗∗∗^	ns	^∗∗∗^	^∗∗∗^	ns
	(mg g^–1^)	N10	33.68 ± 0.33	14.50 ± 0.57	7.32 ± 0.26	4.77 ± 0.33	14.80 ± 0.44						
*Hemarthria altissima*	Proline	N0	0.04 ± 0.01	0.07 ± 0.01	0.11 ± 0.01	0.15 ± 0.01	0.09 ± 0.01	^∗∗∗^	^∗∗∗^	^∗∗∗^	^∗∗∗^	^∗∗∗^	ns
	(μmol g^–1^)	N10	0.17 ± 0.01	0.21 ± 0.02	0.31 ± 0.02	0.46 ± 0.02	0.23 ± 0.01						
	Soluble sugar	N0	32.87 ± 3.36	38.36 ± 0.65	47.06 ± 1.45	57.70 ± 2.05	32.90 ± 2.12	^∗∗∗^	^∗∗∗^	^∗∗^	ns	^∗∗∗^	ns
	(mg g^–1^)	N10	45.38 ± 6.55	64.10 ± 1.62	72.57 ± 3.11	90.81 ± 3.86	49.15 ± 1.78						
	Starch	N0	67.85 ± 0.55	47.03 ± 3.85	25.86 ± 4.56	15.99 ± 1.65	58.69 ± 4.60	^∗∗∗^	^∗∗∗^	^∗∗∗^	ns	^∗∗∗^	^*^
	(mg g^–1^)	N10	44.85 ± 1.94	43.88 ± 1.59	16.76 ± 0.86	18.05 ± 0.96	47.74 ± 3.84						
*Leymus chinensis*	Proline	N0	0.11 ± 0.01	0.12 ± 0.01	0.15 ± 0.01	0.17 ± 0.02	0.16 ± 0.01	^∗∗∗^	^∗∗∗^	^∗∗∗^	^∗∗∗^	^∗∗∗^	^∗∗∗^
	(μmol g^–1^)	N10	0.32 ± 0.014	1.24 ± 0.05	4.46 ± 0.14	7.29 ± 0.09	1.91 ± 0.03						
	Soluble sugar	N0	70.99 ± 1.31	53.71 ± 3.41	48.97 ± 2.04	40.65 ± 3.41	55.05 ± 4.04	^∗∗∗^	^∗∗∗^	^∗∗∗^	^∗∗∗^	^∗∗∗^	^∗∗^
	(mg g^–1^)	N10	104.26 ± 3.55	63.59 ± 3.09	45.54 ± 1.97	42.90 ± 1.92	72.92 ± 2.91						
	Starch	N0	26.47 ± 0.17	22.70 ± 1.30	17.70 ± 1.10	18.71 ± 1.24	25.60 ± 2.07	^∗∗∗^	^∗∗^	^∗∗∗^	^∗∗∗^	^∗∗^	^∗∗^
	(mg g^–1^)	N10	37.71 ± 2.50	14.53 ± 0.87	14.70 ± 0.96	15.10 ± 1.23	24.30 ± 1.14						

Results of two-way analysis of variance on the effects of drought (D), N addition (N) and their interactions (D **×** N), as well as re-watering (R), N and their interactions (R **×** N) on the content of proline, soluble sugar and starch are provided. Levels of significance are indicated as: ns – not significant, ^*^P < 0.05, ^∗∗^*P* < 0.01, and ^∗∗∗^*P* < 0.001. Data are reported as the arithmetic mean ± 1 standard error (*n* = 6).

**FIGURE 6:**
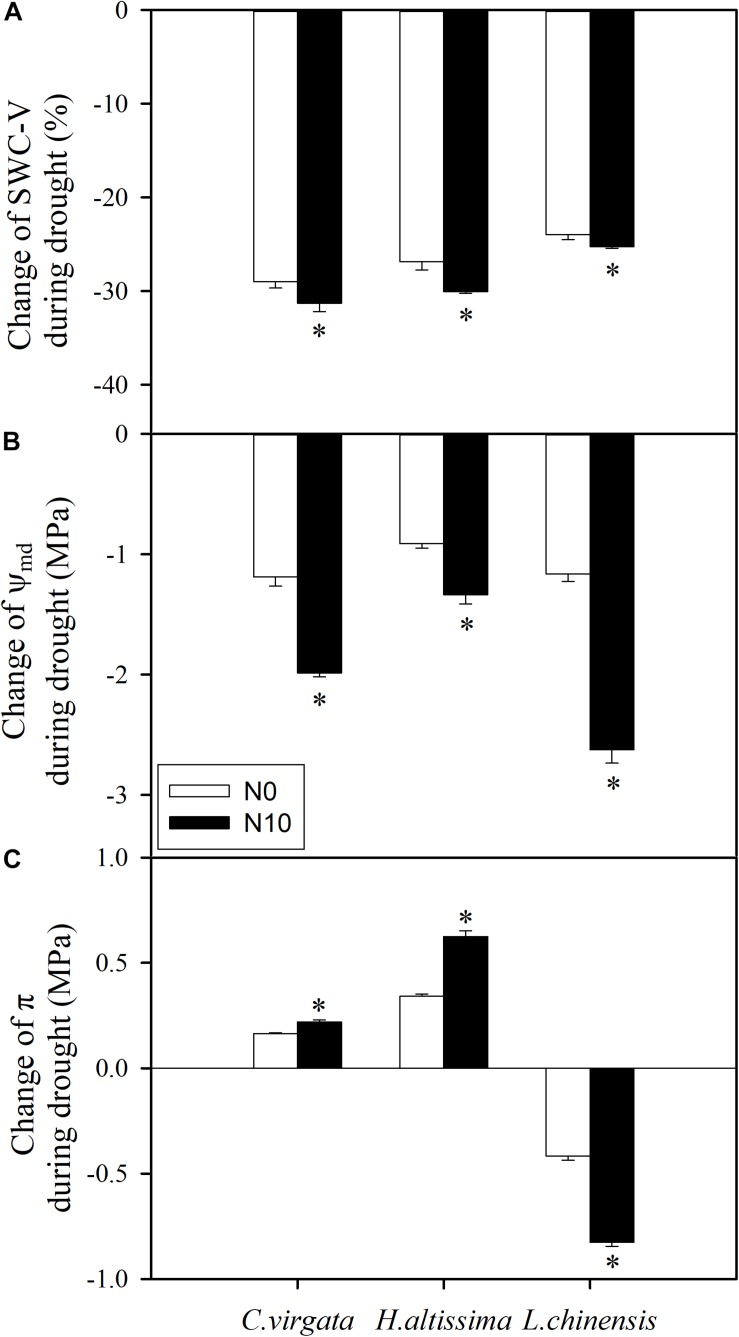
Drought induced changes in **(A)** soil water content (SWC-V, %), **(B)** leaf mid-day water potential (Ψ_md_; MPa), and **(C)** leaf osmotic pressure (π; MPa) in *C. virgata* (annual C_4_), *H. altissima* (perennial C_4_), and *L. chinensis* (perennial C_3_) under unfertilized (N0) and fertilized (N10) conditions. “^*^” represents significant differences between the N treatments (*P* < 0.05). Data are reported as the arithmetic mean ± 1 standard error.

Soil water content was reduced by 31 ± 0.90% and there was a reduction of midday water potential (Ψ_md_) from −1.48 ± 0.07 to −3.46 ± 0.08 MPa for the fertilized pots with *C. virgata* (Figures 1D, 6A). Assuming the plants were in equilibrium with the soil, this corresponds to 30% of the overall 2 MPa change due to volume reduction, or 0.6 MPa. The resulting 1.3 MPa difference in Ψ_md_ during drought is likely due to the production of osmotically active compounds, and Figure 6 shows that osmotic pressure (π) increased by 114 ± 3.9% during drought for the fertilized *C. virgata*. Using the van’t Hoff relation (Nobel, 2001), we note that the change in concentration of proline during drought contributes about 0.026 MPa to the overall change in Ψ for the fertilized *C. virgata*, 0.001 MPa for the fertilized *H. altissima*, and 0.018 MPa for the fertilized *L. chinensis*. Likewise, the change in sugar concentrations in the unfertilized plants from day 1 to 7 for *C. virgata* corresponds to 0.39 ± 0.01 MPa, a doubling to 1.25 ± 0.04 MPa for *H. altissima*, and a decrease in π from 1.43 ± 0.03 to 0.59 ± 0.02 MPa for *L. chinensis* (Figure 6C).

### Antioxidant Enzyme Activities

**FIGURE 7:**
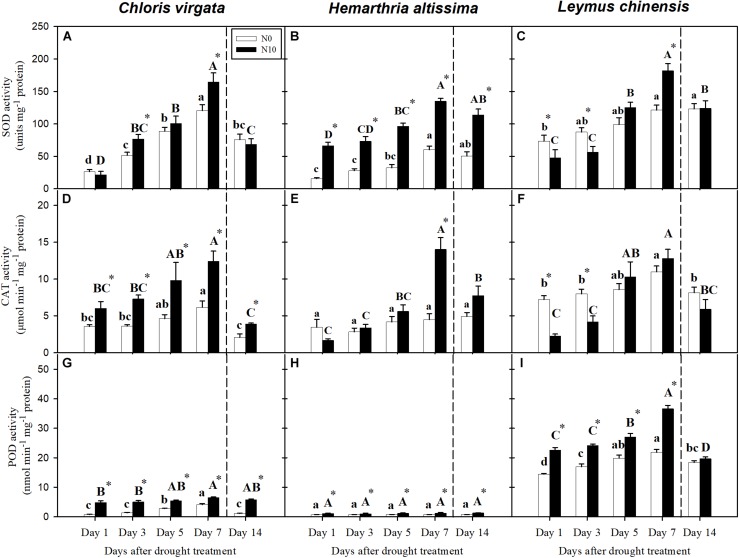
Activities of **(A–C)** superoxide dismutase (SOD: units mg^–1^ protein), **(D–F)** catalase (CAT: μmol min^–1^ mg^–1^ protein), and **(G–I)** peroxidase (POD: nmol min^–1^ mg^–1^ protein) in *C. virgata* (annual C_4_), *H. altissima* (perennial C_4_), and *L. chinensis* (perennial C_3_) on days 1, 3, 5, 7, and 14 of the drought/re-watering treatment. Different lower-case letters and capital letters indicate significant differences among the measuring dates under the unfertilized (N0) treatment and the fertilized (N10) treatment, respectively. “^*^” represents significant differences between the N treatments (*P* < 0.05). Data are reported as the arithmetic mean ± 1 standard error (*n* = 4). Vertical dashed lines denote the separation of drought and re-watering period.

With the intensification of drought stress, the antioxidant enzyme activities (SOD, CAT, and POD) were upregulated (Figures 7A–G,I), except for POD in *H. altissima* (Figure 7H). In general, N-fertilized grasses had the highest antioxidant enzyme activities, especially at the end of the drought period (Figure 7). At the end of the re-watering period, greater SOD activities were detected in all grass species compared to the pre-drought values (Figures 7A–C). For CAT activities, no significant differences were observed between day 14 and day 1 in *C. virgata* and *L. chinensis*. Compared to the values on day 1, significant differences in POD activities were detected only in *L. chinensis* on day 14 (Figure 7I).

## Discussion

### Resistance of Photosynthesis to Water Stress

Our results demonstrated that a sharp reduction in soil moisture during the drought experiment (Figures 1A–C) and a significant downregulation of photosynthesis for both of the representative C_3_ and C_4_ grasses from northeastern China (Figures 2A–C). Consistent with previous studies, stomatal closure was an early response to drought and an effective way to reduce water loss (Figures 2D–I); however, it also limits CO_2_ diffusion into the leaves, which causes the decrease in *A* (Cornic et al., 2000). Under drought conditions, plants tend to maintain or increase WUE to cope with water limitation (Ripley et al., 2010). However, this was observed only in the C_4_ grasses from day 1 to day 5 under the drought treatment (Figures 2J–O). Contrary to our hypothesis, C_4_ grasses maintained high WUE under drought conditions and thus maintained their photosynthetic advantage relative to C_3_ grasses (Taylor et al., 2014). As drought stress further intensified, the severe reduction in *A* (Figures 2A–C) resulted in dramatic declines in *A*/*g*_s_ and *A*/*E* in both the C_3_ and C_4_ grasses (Figures 2J–O). Nitrogen addition increased the N content of leaves (Supplementary Figure 3) and thus could enhance their photosynthetic capacity, which could further improve WUE in both the C_3_ and C_4_ grasses, but only when the plants were subjected to drought stress from day 1 to day 5 (Figures 2J–O). This is consistent with the results of previous studies (Niu et al., 2005, 2008b). The positive effects of N addition on WUE disappeared at the end of the drought treatment, which may be attributed to greater biomass (Supplementary Figure 4) and more water consumption by transpiration associated relatively severe drought stress as compared to the unfertilized plants (Figures 1D–F). The results of this study, as well as from many others, suggest that improvement in WUE is an important drought resistance strategy, but it works only under mild drought stress (Ripley et al., 2010).

As has been shown for many C_3_ and C_4_ grasses, drought decreased *A* through a combination of stomatal and metabolic limitations (Lawlor and Cornic, 2002; Ghannoum et al., 2003; Flexas et al., 2006), but the magnitude of these responses differed in these C_3_ and C_4_ grasses, and changed as drought progressed (Ripley et al., 2010). In addition, *g*_m_ (mesophyll conductance for CO_2_ diffusion from the intercellular space to the chloroplast stroma) also plays an important role in limiting photosynthesis (Miyashita et al., 2005). With the further intensification of the drought stress over time, stomatal limitations in the C_4_ grasses (Figures 5A,B) were lower than stomatal limitations in the C_3_ grass species (Figure 5C). Meanwhile, metabolic limitations were dominant at the end of drought period (Figures 5D–F), which indicates that the nature and timing of photosynthetic downregulation were different among these C_3_ and C_4_ grasses. N addition increased leaf N content (Supplementary Figure 3), improved the activities of photosynthetic enzymes (Table 1), and reduced stomatal limitations in the fertilized *C. virgata* and *L. chinensis* plants (Figures 5A,C), which could be attributed to differences in photosynthetic type and life forms. By contrast to the stomatal limitations, there were higher metabolic limitations in the fertilized plants (Figure 5), which may indicate downregulation of Rubisco activity and RuBP regeneration rate due to inadequate ATP or NADPH supply from PSI during water stress (Lawlor, 2002; Parry et al., 2002; Flexas et al., 2004). The decline in photosynthetic capacity (V_cmax_ and J_max_) in the C_3_ grasses and V_cmax_ and V_pmax_ in the C_4_ grasses during drought has been observed in many other grass species (Flexas et al., 2004; Ripley et al., 2010). Therefore, metabolic limitations of photosynthesis in the fertilized grasses (and particularly for the C_3_ grass) changed with N addition treatment, which might alter drought recovery after rehydration. N addition could result in a shift in photosynthetic limitation with the intensified drought stress, resulting in the lower *F*_V_/*F*_M_ (Figure 4) and the higher *PLA* (Figure 3A) in the fertilized plants.

Osmotic solute concentrations and antioxidant enzyme activities have been considered as crucial factors for plant adaptation to drought, because they sustain tissue metabolic activity and reduce progressive oxidative damage resulting from reactive oxygen species, respectively (Morgan, 1984; Zhang and Kirkham, 1996; Chaves et al., 2003). We found a sharp reduction in SWC during the drought treatment (Figures 1A–C) and the percent change in SWC in the fertilized plants was higher than in the unfertilized plants (Figure 6A), which mainly resulted from more plant transpiration caused by more biomass in the fertilized pots (Supplementary Figure 4). The percent change of water potential in the fertilized plants was higher by comparison to those in the unfertilized plants (Figure 6B). For all grass species, as drought stress intensified, there was significant upregulation of proline concentrations in the fertilized compared to unfertilized plants (Table 2), which could improve the stabilization of sub-cellular structures (Ashraf and Foolad, 2007). Plants also use several sugar-based strategies to tolerate environmental stresses (Anderson and Kohorn, 2001), which can also contribute to changes in osmotic pressure in cells (Nobel, 2001). Soluble sugar content tended to increase in the leaves of droughted plants, despite the decreased rates of carbon assimilation in the C_4_ grasses (Figures 2A,B). Notably, the proportional decrease in starch content (e.g., by about 75% from day 1 to 7 for unfertilized *C. virgata*, Table 2) is greater than the increase for sugars (*ca.* 2 ×), indicating that some of the products of starch hydrolysis were respired or translocated to other tissues. However, this was not observed in the C_3_ grass *L. chinensis*, for which both sugar and starch decreased (Chaves, 1991) between day 1 and 7 of drought (Table 2). These results are consistent with our previous study of drought impacts on dark respiration in these three species, in which starch content decreased both in the C_3_ and C_4_ grasses and sugar content increased in the C_4_ grasses but not in the C_3_ grass *L. chinensis* (Zhong et al., 2017).

The osmotic compounds accumulated or synthesized can include anions and cations, amino acids, methylated quaternary ammonium compounds and hydrophilic proteins (Chaves et al., 2003). Certain cellular solutes such as proline and sugars can provide protection of macromolecules, and osmotic adjustment can develop during the drought. We note that the change in concentration and osmotic pressure of proline (Table 2 and Figure 6C) and could conclude that proline likely helps protect these species during the drought period in a non-osmotic manner, such as by stabilizing macromolecules (Reddy et al., 2004). The change in sugar concentrations were different between the C_3_ and C_4_ grasses (Table 2 and Figure 6C). Contrary to the C_4_ grasses, the decrease of π in the fertilized C_3_ grass (Figure 6C) may be due to respiration rather than accumulation of sugars. These results indicate varying degrees of osmotic adjustment during drought among these species, and that some other compounds contribute to the overall change in Ψ during drought. In future studies, other osmotic compounds should be measured to determine the dominant compound(s) of osmotic adjustment in these species.

Superoxide dismutase (SOD), catalase (CAT), and peroxidase (POD) could reduce progressive oxidative damage and ultimately cell death (Smirnoff, 1998; Dat et al., 2000; Sharma et al., 2012). Consistent with previous studies (Zhang and Kirkham, 1996), we detected differences in antioxidant responses to drought in the C_3_ and C_4_ grasses (Figure 5). Presumably these enzymes helped to regulate the excess excitation energy processing within PSII in the absence of endpoint carbon fixation, as indicated by the reductions in *F*_V_/*F*_M_. Nitrogen could enhance the carbon fixation capacity of plants, and is one of the main components of amino acids. The N addition treatment helped upregulate the concentrations of proline and sugars and the activities of antioxidant enzymes in both C_3_ and C_4_ grasses (Table 2). However, N addition could not compensate for the decline in photosynthesis induced by the restriction of photosynthetic enzymes, thus reducing photosynthetic resistance to drought. Yet, such restriction of photosynthetic enzymes would obviate higher antioxidant protection as PSII would continue to be oxidized in sunlit leaves. N addition resulted in greater aboveground biomass, which would likely increase soil water consumption due to increased leaf area and thus transpiration, which resulted in more extreme drought stress (Yahdjian and Sala, 2006; Hu Z. et al., 2010). However, dense canopies can decrease ground-level light availability, thus reducing soil surface temperature and evaporation (Borer et al., 2014). More studies are required for the understanding of N addition impacts on plant water balance and antioxidant enzyme activity.

Extreme drought could lead to photoinhibition, which induces photo-oxidative damage to the photosynthetic apparatus (Valladares and Pearcy, 2002). Dynamic photoinhibition is a reversible, regulatory process that controls the dissipation of excessive light energy for photoprotection by the xanthophyll cycle pool (Demmig-Adams and Adams III, 2000). By contrast, chronic photoinhibition stems from photodamage involving the turnover of proteins D1 and D2 under drought conditions (Werner et al., 2002). Chlorophyll fluorescence measurements (i.e., *F*_V_/*F*_M_) indicate the maximal efficiency of excitation energy capture by “open” PSII reaction centers; decreases in this parameter indicates plants are subjected to photoinhibition during water stress (Souza et al., 2004). The values for *F*_V_/*F*_M_ were lower for all grass species at the end of drought period (Figure 4), consistent with numerous other studies (Ogaya and Peñuelas, 2003; Hu L. et al., 2010; Cardoso et al., 2018). All three species exhibited a two- to sixfold increase in SOD, CAT, and POD enzyme activity. SOD and CAT participate in the de-excitation of the energized xanthophyll cycle (Müller-Moulé et al., 2002), and their increases are consistent with the decreases in *F*_V_/*F*_M_ during drought. *F*_V_/*F*_M_ was significantly lower in the fertilized compared to the unfertilized plants. This suggests greater damage to PSII oxygen-evolving core complexes and reaction centers caused possibly by a lesser ability to adjust thermal dissipation processes in the unfertilized plants (Colom and Vazzana, 2003). For all grass species, the opposite trends between *PLA* and *PRA* in the unfertilized and fertilized plants (Figures 3A,B) reflect to a certain degree dynamic (*PLA*) and chronic (*PRA*) impacts of drought. These are likely due to different photosynthetic limitations and different degrees of damage to PSII at the end of the drought period, which could result in the differences in both time and degree of recovery during the subsequent re-watering period (Galmés et al., 2007b; Hu L. et al., 2010).

### Resilience of Photosynthesis After Re-watering

Analyzing resistance and resilience by assessing photosynthetic limitations during the drought and re-watering periods was an effective method for determining the sensitivity of the C_3_ and C_4_ grasses to changes in soil water availability. While many studies have addressed different aspects of photosynthetic limitations only during drought, the resilience limitations after re-watering have received considerably less attention (Galmés et al., 2007b). The re-watering treatment induced upregulation of photosynthesis, but the patterns of upregulation were different between the C_3_ and C_4_ grasses. Moreover, the extent and rate of recovery was influenced by the N addition treatment (Figures 2A–C, 3B,C), which depended on both the severity of drought stress and the capacity for recovery of stomatal opening and photosynthetic biochemistry (Galmés et al., 2007b; Hu L. et al., 2010; Ripley et al., 2010; Shi et al., 2018). The resilience of photosynthesis suggests that water stress did not irreversibly damage the light reactions of PSII, and that any damage was repaired during re-watering, promoting the recovery of photosynthesis (Flexas et al., 2004). The observed upregulation of *F*_V_/*F*_M_ following re-watering is consistent with this observation (Figures 4A–C), although we note that recovery was not fully complete by day 14.

Normally, the recovery of photosynthesis after a moderate water stress is rapid and almost complete (Flexas et al., 2006). By contrast, after severe water stress, the upregulation of photosynthesis is progressive, slow (taking several days to weeks), and usually incomplete (Miyashita et al., 2005). The unfertilized C_4_ grasses had higher R_ML_ and lower *PRA* relative to the unfertilized C_3_ grass (Figures 3B, 4D–F). Similar results have also been reported in other studies (Ripley et al., 2007, 2010). These results suggest the operation of alternative or additional mechanisms of photosynthetic inhibition and constraints on upregulation of photosynthesis in the C_4_ grasses (Figures 2A–C). Quick recovery from drought stress for C_3_ grasses may enhance their competitiveness relative to C_4_ grasses under the scenarios of altered precipitation regimes. For all three grass species, lower soil content were observed in the fertilized pots by comparison to the unfertilized pots (Figures 1A–C), which resulted in lower *PRA* (Figure 3B). Also, photosynthetic recovery was co-limited by incomplete stomatal opening (Figures 2D–F) and upregulation of metabolic pathways in the fertilized pots (Table 1). Photosynthetic CO_2_ assimilation for the fertilized C_3_ and C_4_ grasses did not fully recover at the end of the re-watering period (Figure 3A). Recovery rate of photosynthesis was greater in the fertilized *H. altissima* and *L. chinensis* plants, but not in the fertilized *C. virgata* plants (Figure 3C), which could be attributed to more water stress injury caused by greater plant transpiration for *C. virgata* (Figure 2G and Supplementary Figure 4). The different resilience between *C. virgata* and *H. altissima* may be attributed to subtype associated different drought sensitivity, *C. virgata* and *H. altissima* belong to PEP-carboxykinase enzyme (PEPCK) subtype and NADP-malic enzyme (NADP-ME) subtype, respectively (Pyankov et al., 2010; Luo et al., 2013). Compared with NAD-malic enzyme (NAD-ME) subtype, NADP-ME, and PEPCK subtypes are less drought resistant under the drought environment (Hattersley, 1992). However, there is no research about the drought sensitivity between PEPCK and NADP-ME subtypes and consistent conclusion about the sensitivity difference among the C_4_ subtypes (Ellis et al., 1980; Hattersley, 1992; Taub, 2000). Moreover, drought sensitivity may differ among subfamilies independent of C_4_ subtype. Taub (2000) reported that Panicoideae is more resistant to drought compare to the Chloridoideae. Eventually, *C. virgata* and *H. altissima* are differed in life form, and have different root system, which may also attribute to the detected different drought sensitivity (Peek et al., 2005). Greater recovery rate of photosynthesis could be because N addition increased leaf N content and enhanced N allocation to Rubisco, which is the primary CO_2_ fixation enzyme and has a low rate of catalysis (Makino et al., 1992; Makino, 2011; Fleischer et al., 2013).

The recovery of photosynthesis was constrained by both stomatal and metabolic limitations (Figures 2D–F and Table 1). Galmés et al. (2007b) found that recovery of *g*_m_ is the most important factor limiting photosynthetic upregulation after severe water stress. The limited resilience of hydraulic conductivity in the leaf is the apparent cause of the reduced stomatal conductance after re-watering (Galmés et al., 2007b), and it has been shown that aquaporins also play an important role in the regulation of dynamic changes in the variable hydraulic conductance in the leaves (Cochard et al., 2007). Recently, Johnson et al. (2018) showed that the water conducting system could be damaged (via xylem cavitation) by drought, and leaves could not recover hydraulic conductivity after rehydration. Prolonged drought would cause more severe damage in the fertilized plants, which could make recovery more difficult after rehydration. The role of *g*_m_ in explaining photosynthetic limitation and the extent of xylem embolism within these grass leaves needs to be further investigated.

Despite the experiment was carefully designed and results are promising, we recognize the limitations of using pot experiments to predict the sensitivity of photosynthesis in C_3_ versus C_4_ grass species in response to climate change (Passioura, 2006; Poorter et al., 2012). Firstly, the temperature was not controlled in this study, which may impact the water and N responses of C_3_ and C_4_ species (Niu et al., 2008a). Secondly, the plant nutrition, water relations, or other interactions in the rhizosphere may be difficult to relate to plants growing in the field, even if field soils are used (Pankhurst et al., 2002). Finally, pot size could influence the structure and physiology of roots, which further limit the plant photosynthesis and growth (Falik et al., 2005; Passioura, 2006). Future research needs to be conducted in natural plant communities with multiple C_3_ and C_4_ species coexisting and concurrent with manipulations of more environmental factors.

## Conclusion

Compared to the C_3_ grass *L. chinensis*, the C_4_ grasses *C. virgata*, and *H. altissima* had greater carbon assimilation rates under moderate drought conditions, but this advantage was lost at the end of the drought treatment, especially for the fertilized grasses. In the initial stage of the drought period, N addition enhanced the activities of photosynthetic enzymes and reduced stomatal limitation of all grass species, which resulted in higher photosynthetic CO_2_ assimilation in the fertilized grasses. However, N addition caused a strong increase in biomass and resulted in more severe drought stress in the later period of the drought treatment, leading to a change in the dominant photosynthetic limitation and greater downregulation of photosynthetic rates. Nitrogen addition caused upregulation of the concentrations of proline and sugars and the activities of antioxidant enzymes, and the reduction in photosynthetic enzyme activity during drought did not fully recover after rehydration. N addition resulted in greater *PLA* under the drought conditions and lower *PRA* at the end of the re-watering treatment. However, N addition resulted in higher N content in the leaves, which led to faster recovery of *A* upon re-watering. The findings of this study indicate that the effect of N addition on photosynthesis during drought was asymmetric, especially in the plants with a low PNUE such as our C_3_ grass species. In order to understand the explicit roles of water and nutrients in regulating the temporal niche separation among plant functional types differing in photosynthetic pathway, future manipulative research needs to be conducted in natural plant communities with multiple coexisting C_3_ and C_4_ species.

## Data Availability

All datasets generated for this study are included in the manuscript and/or the Supplementary Files.

## Author Contributions

SZ, WS, and J-YM conceived and designed the experiments. SZ, BM, and YX conducted the experiments. WS and SZ analyzed the data and wrote the manuscript. ML provided several suggestions and critically reviewed the manuscript. All authors read and approved the manuscript.

## Conflict of Interest Statement

The authors declare that the research was conducted in the absence of any commercial or financial relationships that could be construed as a potential conflict of interest.
