# Supplementary material for: Nitrogen Addition Increases the Sensitivity of Photosynthesis to Drought and Re-watering Differentially in C3 Versus C4 Grass Species
Source: Front Plant Sci. 2019 Jul 3;10:815. doi: 10.3389/fpls.2019.00815 (PMC6616207; doi:10.3389/fpls.2019.00815)

**Nitrogen addition increases the sensitivity of photosynthesis to drought and re-watering differentially in C_3_ versus C_4_ grass species**

Shangzhi Zhong^1^, Yueqiao Xu^1^, Bo Meng^1^ Michael E. Loik^2^, Jian-Ying Ma^3,*^ and Wei Sun^1,*^

^1^ Key Laboratory of Vegetation Ecology, Ministry of Education, Institute of Grassland Science, Northeast Normal University, Changchun, Jilin Province, P. R. China 130024

^2^ Environmental Studies Department, University of California, Santa Cruz, CA 95064, USA

^3^ Key Laboratory of Biogeography and Bioresources in Arid Land, Xinjiang Institute of Ecology and Geography, Chinese Academy of Sciences, Urumqi, P. R. China, 830011

*Corresponding author

**Jian-Ying Ma**

Xinjiang Institute of Ecology and Geography, Chinese Academy of Sciences

Urumqi, 830011, China

Tel.: +86 991 7885425

E-mail address: [jyma@ms.xjb.ac.cn](mailto:jyma@ms.xjb.ac.cn)

**Wei Sun**

Key Laboratory for Vegetation Ecology, Ministry of Education

Institute of Grassland Science, Northeast Normal University

Changchun, Jilin Province, P. R. China 130024

Tel.: +86 431 8509 8187

E-mail address: [sunwei@nenu.edu.cn](mailto:sunwei@nenu.edu.cn)

**Supplementary Figure 1.** Timeline figure indicates the dates and age of plants after germinating under different treatment periods of the whole experiment.


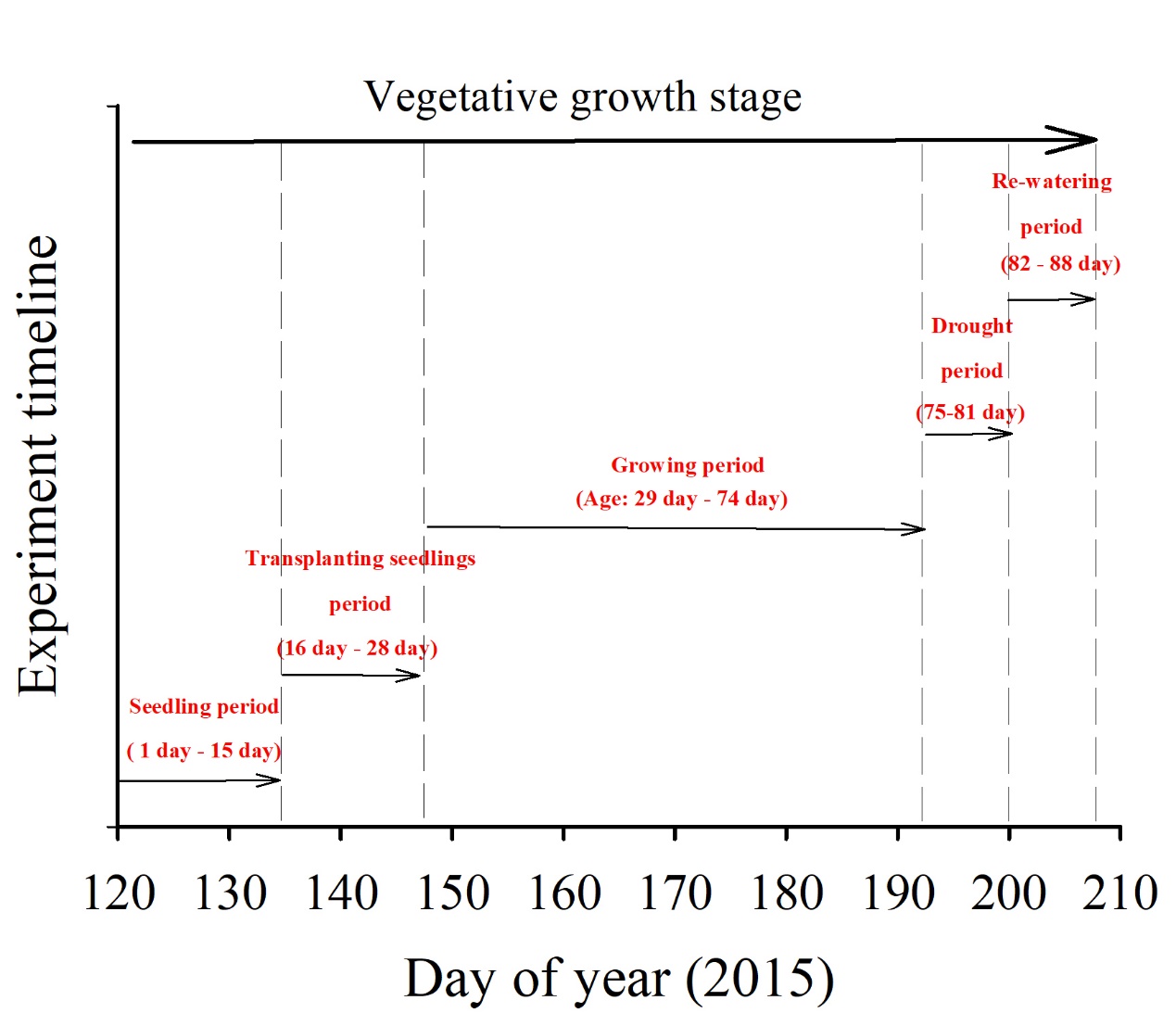


**Supplementary Figure 2.** Meteorological data: (a) diurnal mean air temperature, (b) diurnal photosynthetic photon flux density, (c) diurnal mean air relative humidity and (d) diurnal mean air water vapor pressure on the days 1, 3, 5, 7 and 14 of the drought/re-watering treatment.

**
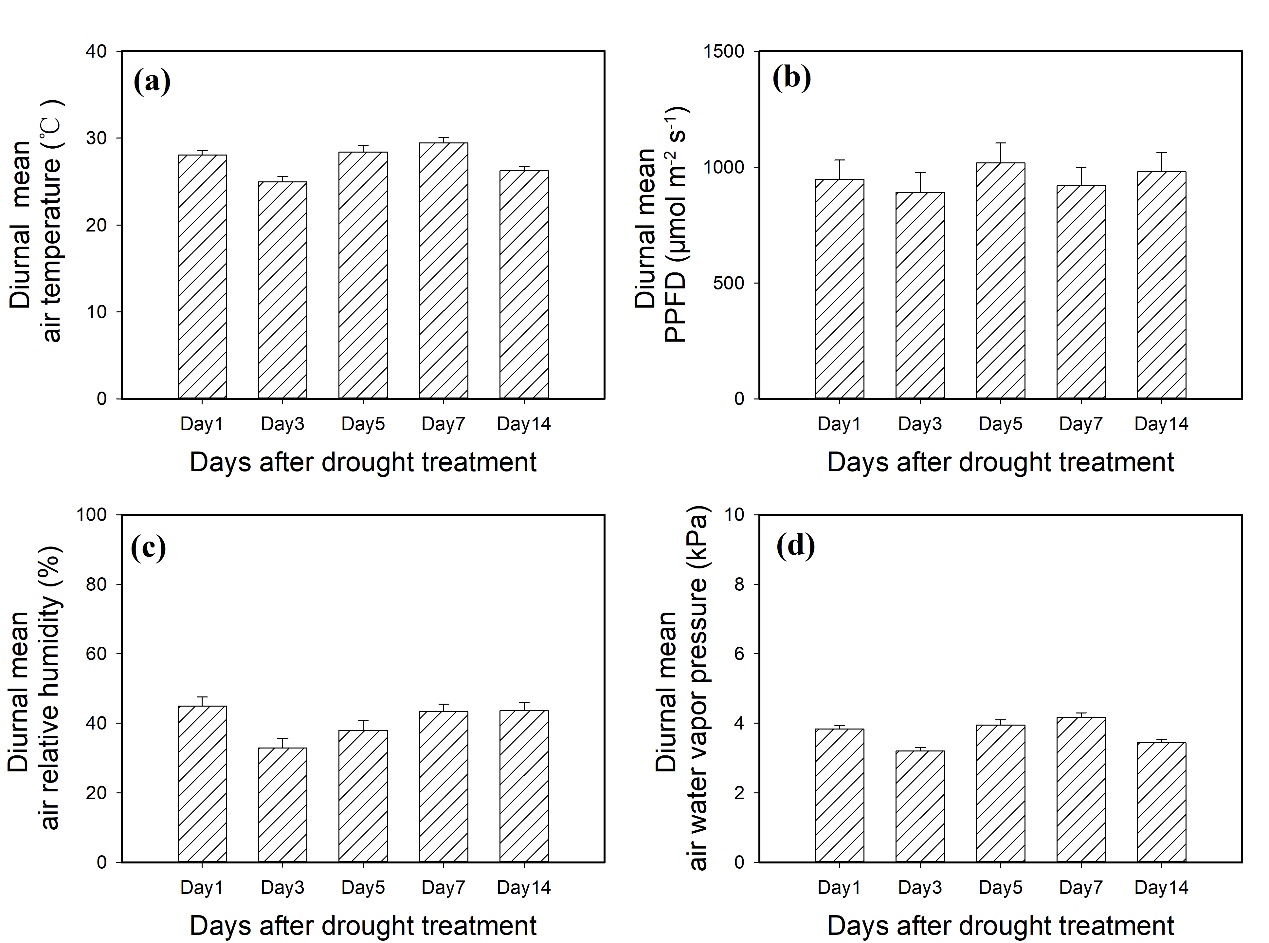
**

**Supplementary Figure 3.** Leaf nitrogen content in *Chloris virgata* (annual C_4_), *Hemarthria altissima* (perennial C_4_) and *Leymus chinensis* (perennial C_3_) grown in the unfertilized (N0) and fertilized (N10) conditions. “*” represents significant differences between the N treatments (*P* < 0.05). Data are reported as the arithmetic mean ± 1 standard error (n=6).

**
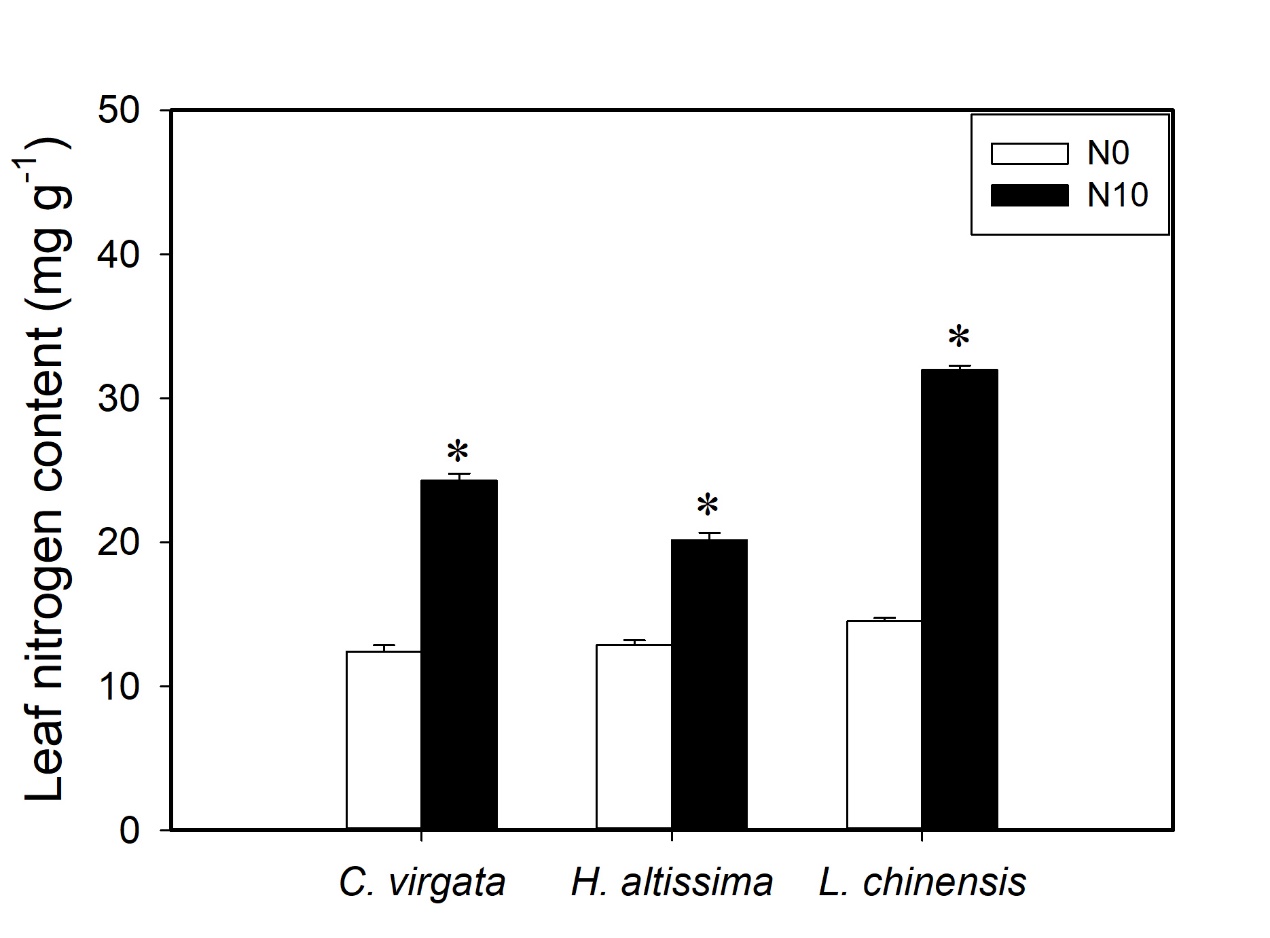
**

**Supplementary Figure 4.** **(a)** Plant height and **(b)** total biomass in *Chloris virgata* (annual C_4_), *Hemarthria altissima* (perennial C_4_) and *Leymus chinensis* (perennial C_3_) grown in the unfertilized (N0) and fertilized (N10) conditions. “*” represents significant differences between the N treatments (*P* < 0.05). Data are reported as the arithmetic mean ± 1 standard error (n=6).


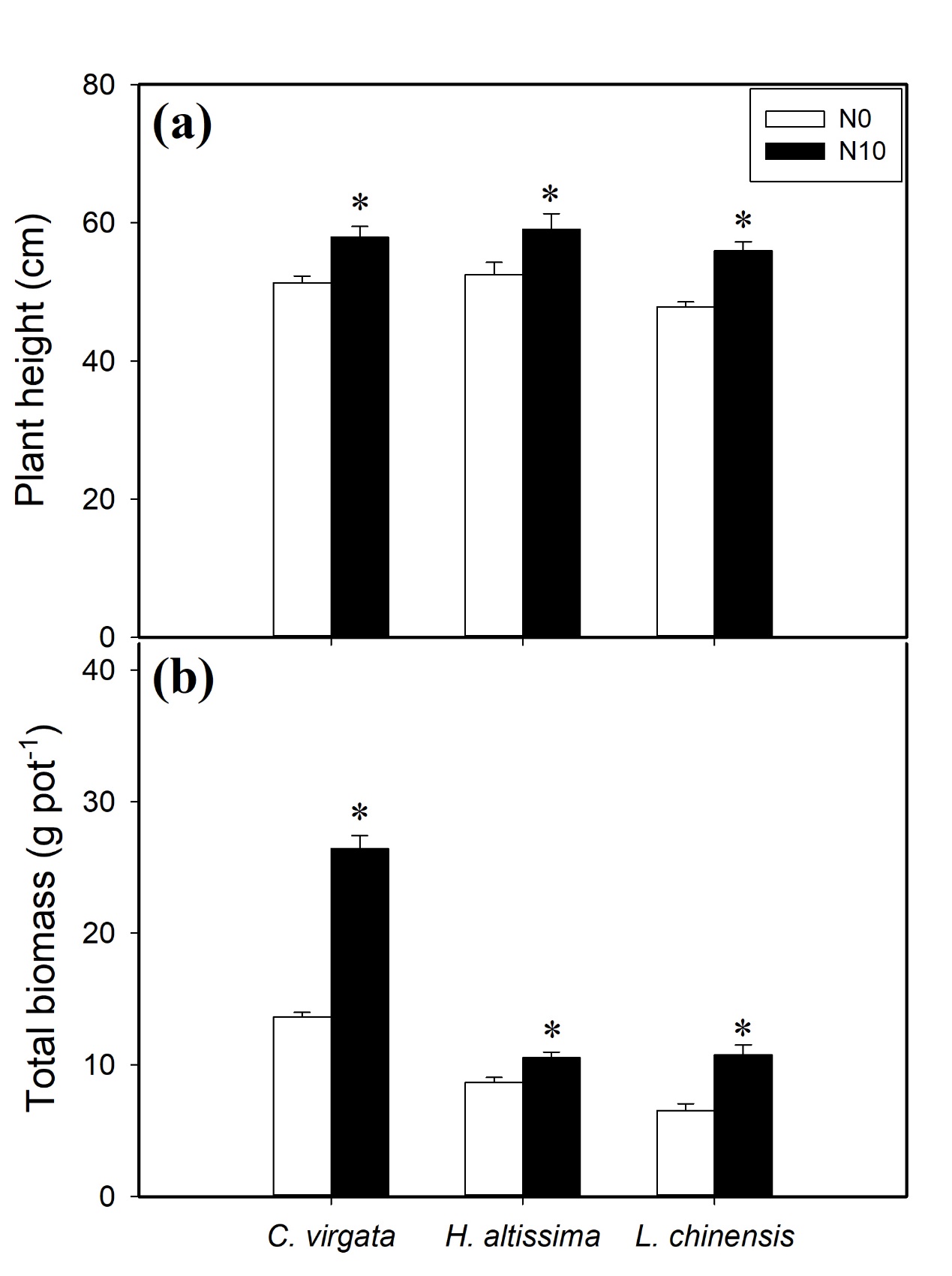

Supplement: Supplementary file 1 [file Data_Sheet_1.docx]
